# Genome Wide Allele Frequency Fingerprints (GWAFFs) of Populations via Genotyping by Sequencing

**DOI:** 10.1371/journal.pone.0057438

**Published:** 2013-03-04

**Authors:** Stephen Byrne, Adrian Czaban, Bruno Studer, Frank Panitz, Christian Bendixen, Torben Asp

**Affiliations:** 1 Department of Molecular Biology and Genetics, Aarhus University, Research Centre Flakkebjerg, Slagelse, Denmark; 2 Department of Molecular Biology and Genetics, Aarhus University, Research Centre Foulum, Tjele, Denmark; 3 Institute of Agricultural Sciences, Forage Crop Genetics, ETH Zurich, Zurich, Switzerland; University of Hong Kong, China

## Abstract

Genotyping-by-Sequencing (GBS) is an excellent tool for characterising genetic variation between plant genomes. To date, its use has been reported only for genotyping of single individuals. However, there are many applications where resolving allele frequencies within populations on a genome-wide scale would be very powerful, examples include the breeding of outbreeding species, varietal protection in outbreeding species, monitoring changes in population allele frequencies. This motivated us to test the potential to use GBS to evaluate allele frequencies within populations. Perennial ryegrass is an outbreeding species, and breeding programs are based upon selection on populations. We tested two restriction enzymes for their efficiency in complexity reduction of the perennial ryegrass genome. The resulting profiles have been termed Genome Wide Allele Frequency Fingerprints (GWAFFs), and we have shown how these fingerprints can be used to distinguish between plant populations. Even at current costs and throughput, using sequencing to directly evaluate populations on a genome-wide scale is viable. GWAFFs should find many applications, from varietal development in outbreeding species right through to playing a role in protecting plant breeders’ rights.

## Introduction

Recent advances in sequencing have improved the potential to characterize genetic variation on a genome-wide scale. Furthermore, this can now be done in species that have little or no pre-existing genomic resources. Current Illumina sequencing instruments are capable of producing up to 3 billion paired-end sequences on a single flow cell (http://www.illumina.com/systems/hiseq_systems.ilmn). To put this into context, it would provide enough data for greater than 100 times coverage of the perennial ryegrass genome (estimated to be 2.69 Gb; [1]) with 100 bp reads. This would still make complete genome re-sequencing costly on a large scale, and unnecessary for many applications. Fortunately, strategies exist to reduce the complexity of genomes to levels that can be sequenced more affordably in studies involving large numbers. These include using probe capture techniques [2], transcriptome re-sequencing [3], and complexity reduction techniques using restriction enzymes [4]. After complexity reduction, samples can be individually tagged to allow multiplexing on a single lane of a flow cell. Cheap barcoding systems have already been developed that enable large-scale multiplexing [5], [6]. The level of multiplexing that can be achieved is only limited by the complexity of the sample and the coverage required, given a specific sequencing throughput.

The genotyping-by-sequencing strategy (GBS) developed by [5] provides a very cheap and simple approach to reduce genome complexity using restriction enzymes that preferentially cut in the low-copy fraction of the genome. There is no random shearing or size selection. This is combined with a simple bar-coding system that adds a short stretch of DNA sequence to one of the sequencing adaptors that is ligated to each DNA fragment. The length of the barcode sequence is varied to ensure that the first 12 bp contains random sequence, which is a requirement for the sequencing software. If barcode lengths were not modulated, all sequences would contain an identical stretch of nucleotides at the same position, corresponding to the restriction enzyme recognition site. Control over complexity reduction is achieved by choosing enzymes that cut at different frequencies in the genome. The choice will ultimately depend on the application and the properties of the genome being studied. A modification to the original GBS protocol has been described recently that employs a two-enzyme digestion, and was applied to linkage mapping in wheat and barley [7].

To date, GBS has only been reported for studies aimed at characterizing genetic variation in single genotypes. There are many applications where characterizing genetic variation in populations will be necessary, for example breeding of outbreeding species. Some previous approaches to characterizing genetic variation in populations have used multi-allelic marker systems, such as Simple Sequence Repeats (SSR) that were typed on pooled DNA [8], [9]. Despite their usefulness, SSR’s are not appropriate for genome-wide analysis requiring large numbers of markers. Large-scale SNP arrays have recently been used to measure allele frequencies in populations [10]–[12], in this case the signal is proportional to the frequency of the allele. However, these SNP arrays are hybridization based, and in outbreeding plant species that have a high diversity there may be inefficiencies in hybridization due to high levels of polymorphism. This may lead to inaccuracies in allele frequency calls. Using GBS would provide a direct way to measure allele frequencies at bi-allelic SNPs throughout the genome. This would enable the generation of fingerprints for a population based on allele frequencies at genome-wide positions. The purpose of this study was to apply GBS for the study of allele frequencies in populations, and determine if the resulting allele frequency fingerprints can be used to distinguish between populations. We have used perennial ryegrass varieties that are populations generated from poly-crosses of multiple genotypes. We tested two restriction enzymes that differ in orders of magnitude in the frequency of cutting sites within the genome and determined the number of loci we would expect to genotype with different sequencing budgets. The experiment was designed for one of the restriction enzymes to allow evaluation of the effect of barcode on the number of reads recoverable for each sample. The reproducibility of the allele frequency fingerprints was evaluated, and our ability to distinguish between varieties based on these fingerprints was investigated.

## Materials and Methods

### Population Sampling

For each perennial ryegrass variety 0.5 g of seed was weighed out and sprinkled onto wet soil packed in to an eight by eight cm square pot. Seeds were covered with perlite and allowed to germinate in growth cabinets. After eight days, the resulting lawn was cut back to 3 cm using a cutting guide, and the cuttings discarded. The bottom 3 cm was then sampled and placed in envelopes, flash frozen and stored at −80°C. This was performed in four replicates for each of the eight perennial ryegrass varieties Sponsor, Stolon, Glenveagh, Mongita, Bronzyn, Beatrice, Chardin, and Greenway. These will be referred to as ‘sampling replicates’. DNA was isolated from ground tissue.

### Library Preparation and Sequencing

Libraries for GBS were generated according to the protocol developed for maize [5]. Libraries were generated using two restriction enzymes, ApeKI (5 bp recognition site) and PSTI (6 bp recognition site). Alternative enzymes were tested for their suitability to generate GBS libraries and the results of this are shown in Methods S1. In the case of ApeKI, eight libraries were created, each consisting of 32 individually bar-coded samples originating from the same variety. Each of the four ‘sampling replicates’ was divided into eight ‘technical replicates’. This was necessary because all fragments will possess the sequence of the enzyme recognition site, and it is therefore important that libraries have a mixture of bar-codes of various compositions and length to ensure the assumption of randomness in the first 12 bp is not violated. Designing the multiplexing with technical replicates allowed us to investigate the impact of bar-code on data yield, as the same set of 32 bar-codes were used to create each of the eight libraries. Each library was sequenced on a single lane of an Illumina GAII instrument to generate single end sequences of 101 bp. In the case of PstI we created one library consisting of 32 individually bar-coded samples. Each of the four ‘sample replicates’ from each of the eight varieties were included in this library. The library was sequenced on a single lane of an Illumina Hi-Seq 2000 instrument to generate single end sequences of 101 bp.

### Data Analyses

Samples were demultiplexed using sabre (https://github.com/najoshi/sabre), allowing no mismatches within the barcode. All reads were trimmed back to 64 bp in response to an observed deterioration of quality towards the end of the reads in some sequencing lanes. Command line tools from the FASTX-Toolkit were used to trim adaptors and quality filter reads (to pass, 95% of a read needed to have Q scores of at least 15). Any reads with less than 64 bp after adaptor trimming were eliminated. We used the program ustacks [13] to obtain a set of consensus sequences representing the loci available for genotyping. We concatenated all processed reads from each sample and ran through ustacks with the deleveraging algorithm switched off, a minimum read depth for stack formation of 3, the maximum distance allowed between stacks was set to 2, and the maximum distance allowed to align secondary reads to primary stacks was set to 4. Reads were mapped back to consensus loci using bowtie [14] allowing two mismatches across the entire read (-v 2) and only considering uniquely mapped reads (-m 1). Samtools sort and view [15] were used to generate sorted bam files and mpileup was used to generate a pileup. VarScan [16] was used to call variants from the pileup, which was processed and filtered using awk commands as required for the various analysis performed. All statistical analysis and graphics were generated in R [17].

## Results

### Sequencing of GBS Libraries

The sequencing of the eight ApeKI GBS libraries on the Illumina GAII instrument yielded between 24 and 28 million sequences per lane, prior to any processing. In a first analysis we demultiplexed all libraries and counted the number of reads assigned to each sample. The Coefficient of Variation (CV) for reads per sample varied between 0.27 and 0.33 for the eight libraries. The design of the ApeKI experiment, using the same 32 barcodes in each of the 8 lanes, allowed us to evaluate the effect of barcode length on the number of reads recovered. There was a significant effect of both barcode, F(31, 223)  = 20.93, *p*<0.001, and barcode length, F(4, 250)  = 29.89, *p*<0.001 on read numbers. This effect of barcode length limits our ability to cluster barcodes into groups of equal efficiency, due to the need to modulate barcode length. Results S1 contains detailed information on read number per barcode and sample. After quality filtering, 104 million reads remained for analysis.

The sequencing of the library generated by PstI digestion on the Illumina HiSeq 2000 instrument yielded over 130 million sequences from the single lane. The CV for reads per sample after demultiplexing was 0.62 (see Results S1). However, following removal of three clear outliers, the CV dropped to 0.36. After quality filtering, 67 million reads remained for analysis. All sequence data from both ApeKI and PstI libraries have been submitted to the European Bioinformatics Institute (EBI) Short Read Archive (SRA) with the study accession number ERP002166.

### How Many Loci do we Expect to Genotype?

GBS produces reads that fall into discrete piles representing unique loci. The number of loci generated will depend on the frequency of the restriction enzyme recognition site within the genome, and on methylation patterns (in the case of methylation sensitive enzymes). The two enzymes tested in this study differ in the frequency of their recognition site within the genome. We used the sequenced genome of maize, which is a similar size to perennial ryegrass, to perform *in silico* digestions. ApeKI generated over 2.2 million fragments between 100 and 1000 bp, while PstI generated over 136 thousand fragments between 100 and 1000 bp (methods S1). Although this does not take methylation patterns into consideration, it does give an indication on the expected efficiency of the genome complexity reduction.

In order to identify a non-redundant set of loci we used the ustacks algorithm on the complete data set for both enzymes. The number of loci generated was 1,955,745 for ApeKI, and 252,879 for PstI. Therefore, based on our data and ustacks settings, there is nearly eight times as much loci available for genotyping when using ApeKI compared to PstI. If the likelihood of sequencing each locus was uniform, ApeKI libraries would require much greater levels of sequencing to ensure genotyping of a common set of loci across all samples with sufficient coverage, which will be particularly important for accurate determination of allele frequencies. However, all loci are not sequenced uniformly, leading to some loci being sequenced far more frequently than others. This is due to enrichment of shorter fragments during the PCR amplification step, and the more efficient bridge amplification of shorter fragments during sequencing on an Illumina flow cell.

**Figure 1:**
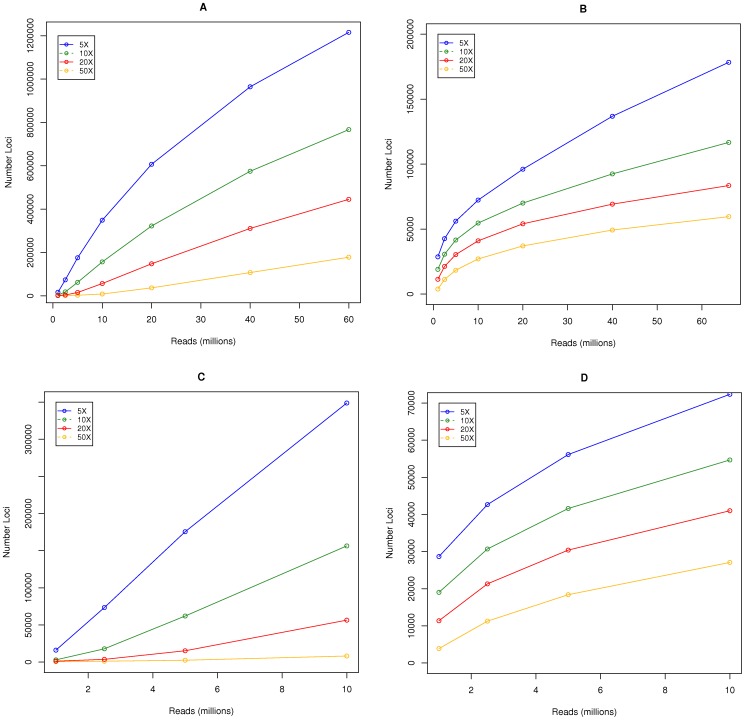
Genotyping potential using different restriction enzymes. Estimating the number of loci we can potentially genotype after genome complexity reduction with (a) ApeKI, and (b) PstI., Plots zoomed in on low sequencing budgets are shown in (c) ApeKI and (d) PstI. Reads were randomly sampled to represent different sequencing budgets, and mapped back to the consensus loci that were generated by merging reads into matching stacks with ustacks. The number of loci achieving a 5, 10, 20, and 50X coverage are shown. Note: the scale of y-axis differs between plots.

In order to establish the number of loci we can expect to genotype given different sequencing budgets, we randomly sampled reads (between 1 and 60 million) and mapped them back to the set of consensus loci generated by ustacks. We then determined the number of loci achieving a 5, 10, and 20 X coverage with the different sequencing budgets. The results are plotted in Figure 1a and 1b. As we increase our sequencing budget for libraries generated with ApeKI, we get a rapid increase in the number of loci genotyped at a 5, 10 and 20X coverage. At relatively low sequencing budgets (e.g 1 million reads per sample) we would expect to genotype more loci with PstI compared to ApeKI. However, as we increase the sequencing budget, ApeKI quickly outperforms PstI in terms of the number of loci genotyped. At a sequencing budget of 2.5 million reads per sample we would expect to genotype more loci to a 5X coverage with ApeKI.

Ensuring that a common set of loci is genotyped across samples requires a strong correlation in the coverage levels of individual loci. Pearson correlation analysis of coverage at loci containing the selected SNP sites (identified with MAF of 5%, see below) for PstI and ApeKI libraries was performed between samples and clearly demonstrates a high correlation in the coverage at each locus (Figure S1). This confirms that loci sequenced a relatively large number of times in one sample will be the same loci that are sequenced a relatively large number of times in all other samples.

### SNP Harvest

Each perennial ryegrass variety is generated from a polycross of multiple parents, and the resulting populations will possess numerous haplotypes (in excess of 20 in many cases). In order to get an idea of the potential SNP harvest across the eight varieties for each restriction enzyme, we pooled together an equal number of reads from each variety. In the case of the ApeKI libraries, 12 million quality filtered reads were pooled from each variety, and in the case of the PstI libraries, 4 million quality filtered reads were pooled for each variety. Ensuring an equal contribution from each variety was done to obtain more accurate estimates of allele frequencies across the eight varieties. The pool of reads was mapped against the consensus loci, and the Minor Allele Frequency (MAF) was estimated from the read coverage. MAF thresholds of either 1 or 5%, and a minimum of 5 reads supporting each allele was required to call a bi-allelic SNP. Distribution of SNPs within reads showed that there was no bias towards SNP identification in the 3′ end of reads for PstI libraries, and only a slight increase in the number of SNPs found in the last 10–15 bp for ApeKI libraries (Figure S2). Using a MAF of 5% we identified in excess of 40,000 SNPs using PstI, and in excess of 640,000 using ApeKI (Table 1). Many individual loci contained multiple SNPs (Figure S3). These positions became our reference SNP panel for calling allele frequencies in individual samples. To do this we mapped quality filtered reads from each of the 32 samples of both GBS libraries onto their corresponding consensus loci and filtered for positions matching the reference SNP panel. The number of reads per sample after quality filtering ranged between 2.7 and 4.5 million per sample for ApeKI libraries, and between 1.2 and 7.2 million per sample for PstI libraries (after removal of three poorly represented samples). We then determined the number of SNPs that we could genotype at different levels of coverage between 5 and 50X, in at least 75% of our samples (Table 2). Not surprisingly, as we increase the minimum coverage threshold required to make a genotype call, the number of SNPs we are able to genotype in at least 75% of the samples drops accordingly.

**Table 1:** Number of SNPs identified using a pool of equal numbers of quality filtered reads from each of eight varieties, totaling 96 million for ApeKI libraries, and 32 million for PstI libraries.

Enzyme	Minimum MAF	No. SNPs	Non redundant loci with SNPs
**PstI**	1%	52,020	27,593
	5%	40,621	24,583
**ApeKI**	1%	780,204	368,915
	5%	643,498	354,988

SNPs were identified using a Minor Allele Frequency (MAF) of 1 or 5 percent.

**Table 2:** The number of SNP positions from the reference panel that was identified in the pooled data set, which could be genotyped in at least 75 percent of individual samples with increasing minimum coverage thresholds.

Enzyme	No. SNPs predicted at MAF 5%^a^	Coverage	SNPs genotyped in 75% of samples^b^
		5X	18,100
		10X	14,527
**PstI**	40,621	15X	12,424
		20X	10,870
		25X	9,593
		50X	5,939
		5X	80,902
		10X	27,067
**ApeKI**	643,798	15X	12,380
		20X	7,328
		25X	5,168
		50X	1,975

a Reference SNP panel identified with a MAF of 5 percent in data pooled from equal numbers of reads of all eight varieties.

b 32 samples for ApeKI libraries, and 29 for PstI libraries (three samples were removed due to very low sequencing coverage).

### Accuracy of Allele Frequency Calls

**Figure 2:**
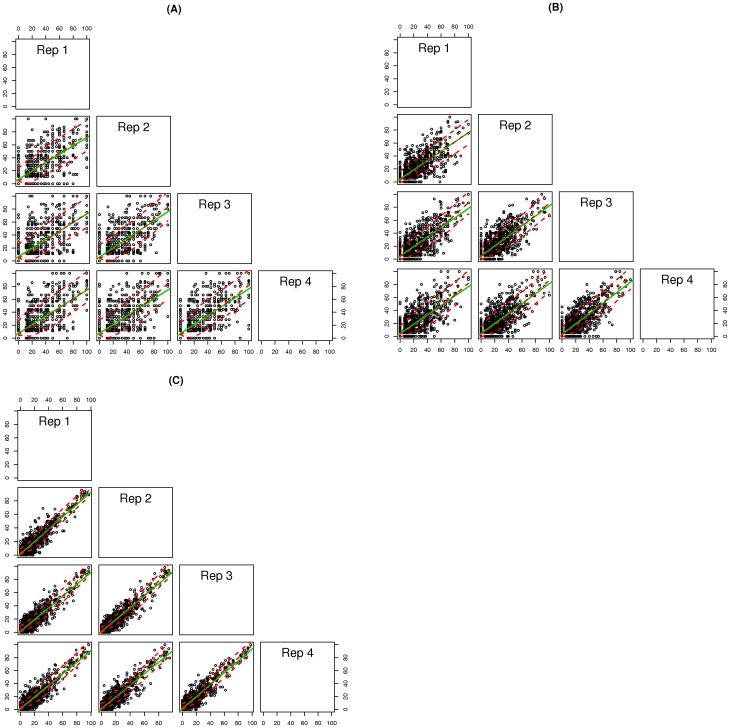
Correlation between allele frequency estimates. Scatter plot matrices between ‘sample replicates’ from the variety Beatrice of the frequency of the variant allele at 1000 randomly selected SNP positions from each of three groupings (a) SNPs with coverage between 5 and 10X in all samples, (b) SNPs with coverage between 10 and 20X in all samples, and (c) SNPs with coverage greater than 20X in all samples. The x and y axis show the frequency of the variant allele. Least squares regression line is shown by solid green line, and Loess smooth is shown by broken red line.

Accurate determination of allele frequencies in a population will be important for many applications and will depend on both good sampling procedures and sequence coverage. We performed four ‘sampling replicates’ for each variety to allow testing of the reproducibility of our sampling protocol. This also enabled us to compare allele frequency calls between replicates using various coverage thresholds. If we can determine allele frequencies accurately, we should see a high level of reproducibility in our allele frequency calls between the ‘sampling replicates’. To investigate this we used data from the ApeKI libraries and calculated the frequency of the variant allele in all samples at the SNP positions identified with a MAF threshold of 5% (Table 2). We looked at all the pairwise combinations of ‘sampling replicates’ for each variety. To investigate the effect of coverage on reproducibility, we placed SNPs into three groups depending on the coverage at this position falling into one of three ranges (5≤X<10, 10≤X<20, X≥20) for each of the four ‘sample replicates’. We randomly sampled 1000 SNP positions from each range to ensure an equal number of data points between comparisons. Scatter plots of all the possible pairwise comparisons for the variety Beatrice are shown in Figure 2. Scatter plots for the remaining varieties showed identical trends and are shown in Results S2. It is clear from the scatter plots that as we increase coverage we improve the reproducibility of our allele frequency calls. The average pearson correlation values of all pairwise comparisons was 0.67 (stdev: 0.01) with coverage levels between 5 and 10, 0.76 (stedv: 0.02) with coverage levels between 10 and 20, and 0.91 (stdev: 0.01) with coverage levels greater than 20.

### Distinguishing Populations Based on Genome Wide Allele Frequency Fingerprints (GWAFFs)

**Figure 3:**
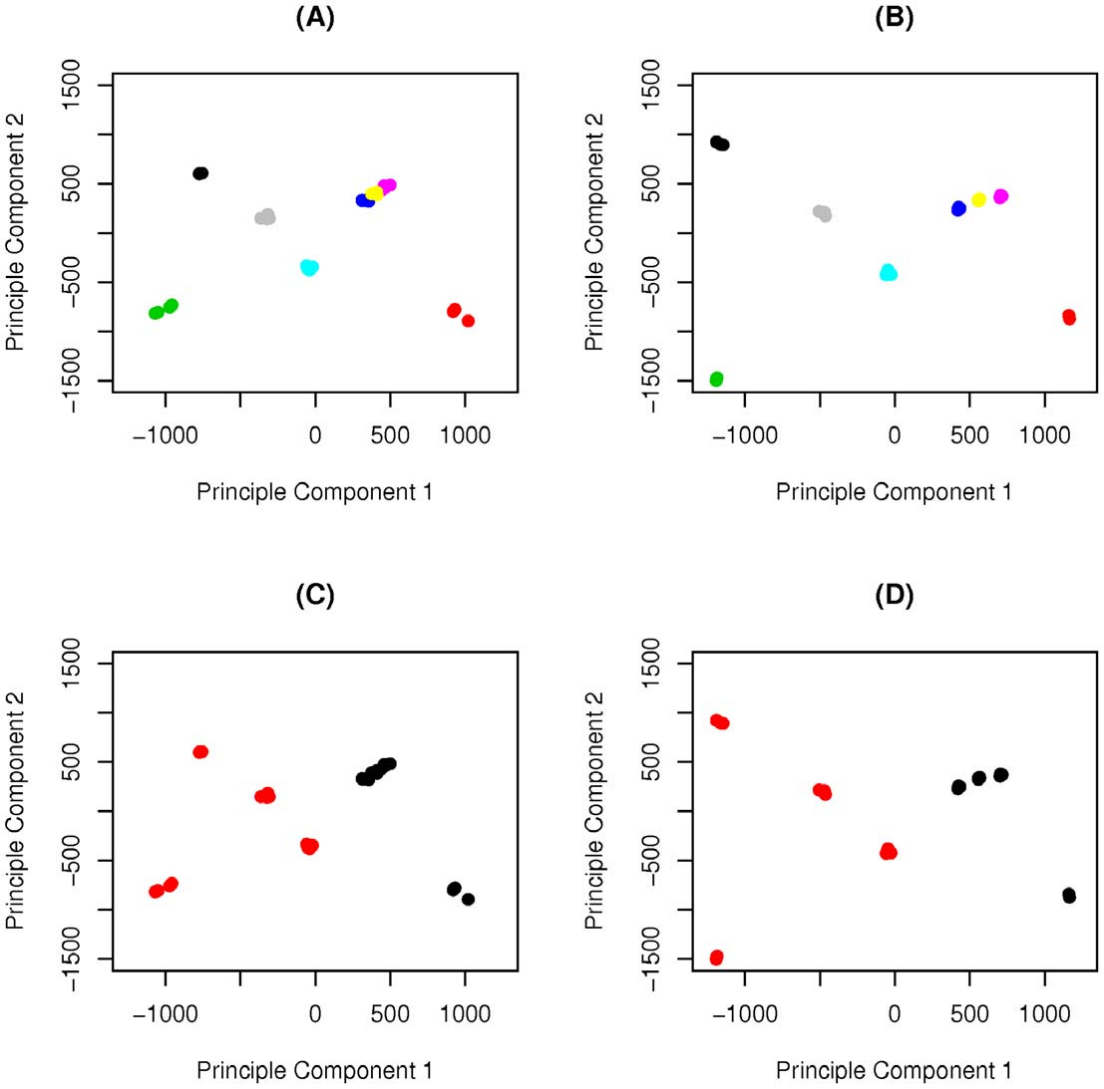
Distinguishing populations based on GWAFFs. Principle Component Analysis (PCA) of GWAFFs based on SNP positions having a minimum coverage of 5 in all samples. (A) and (C) GWAFFs based on allele frequencies of the variant allele at 21,942 SNP positions in ApeKI, (B) and (D) GWAFFs based on allele frequencies of the variant allele at 10,958 SNP positions in PstI. Colours in A and B correspond to the varieties; Beatrice (black), Chardin (Green), Stolon (grey), Greenway (sky blue), Glenveagh (dark blue), Sponsor (yellow), Mongita (purple), and Bronzyn (red). Colours in C and D correspond to type; forage (black), turf (red).

Principle Component Analysis (PCA) was performed to test our ability to distinguish populations, in this case perennial ryegrass varieties, on the basis of GWAFFs. Allele frequencies of the variant allele were determined at reference SNP sites identified with a minimum MAF threshold of 5 percent. For PCA, we only included SNP positions where we were able to call allele frequencies in all samples (32 samples in the case of ApeKI, and 29 in the case of PstI). At a minimum coverage threshold of 5, allele frequencies were determined at 21,942 and 10,958 SNP positions for ApeKI and PstI libraries, respectively. It is important to note that although we applied a minimum coverage threshold of 5, the actual coverage at many SNP positions will be much higher, particularly for PstI which is sampling a much smaller portion of the genome. In both cases the ‘sample replicates’ clustered together, and populations can be distinguished from each other (Figure 3(A) and (B)). In PstI we get a clearer separation of populations, despite only half the number of SNP positions. Again, this can be explained by the mean coverage at each SNP position being much higher in PstI due to greater genome complexity reduction.

The eight varieties used in the study are split equally between forage and turf type. Forage population types are bred primarily for good vegetative growth and high digestibility, whereas turf population types are bred primarily for a fine leaf and dense sward. In Figure 3(C), and 3(D) the samples have been labeled according to type and we can see a split left and right of 0 on the first Principle Component (PC). PC1 explains 20 and 14 percent of the variation in the data for PstI and ApeKI respectively, based on SNP positions with a minimum coverage threshold of 5.

**Figure 4:**
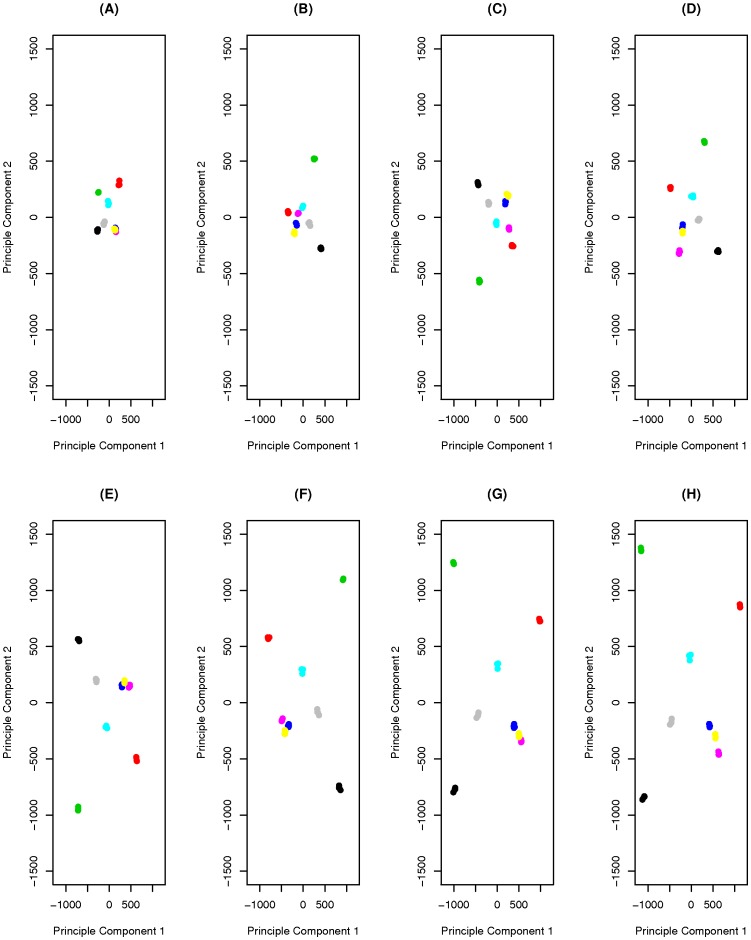
Ability to distinguish between populations using varying numbers of SNPs. Principle Component Analysis (PCA) of GWAFFs based on SNP positions having a minimum coverage of 5 in all samples of PstI library. PCA was performed using a series of randomly selected SNP positions to evaluate the effect of the number of SNP positions on ability to distinguish between populations; (A) 500, (B) 1000, (C) 1500, (D) 2000, (E) 4000, (F) 6000, (G) 8000, and (H) 10000. Colours correspond to the varieties; Beatrice (black), Chardin (Green), Stolon (grey), Greenway (sky blue), Glenveagh (dark blue), Sponsor (yellow), Monigta (purple), and Bronzyn (red).

To determine the effect of the number of SNP positions on our ability to distinguish populations based on GWAFFs, we randomly selected between 500 and 10,000 SNP positions from the PstI libraries. These SNP positions were drawn from the 10,958 that had at least a coverage of 5 in all samples. PCA was performed with each sample size and the results are plotted in Figure 4. At only 500 SNP positions, it becomes difficult to clearly distinguish all populations, chiefly for the three most similar populations based on GWAFFs, Mongita, Sponsor, and Glenveagh (Figure 4(A)). As the number of SNP positions on which the GWAFFs are based increases, our ability to distinguish populations improves (Figure 4(A)–(H)).

## Discussion

The genotyping-by-sequencing (GBS) method described by [5] is a simple and robust genotyping approach for genome-wide analysis. We investigated the utility of using this approach for the determination of allele frequencies in populations. These Genome Wide Allele Frequency Fingerprints (GWAFFs) have numerous applications in both basic and applied research.

### Choosing a Genome Complexity Reduction Strategy

Controlling the extent of genome complexity reduction is achieved by choosing different restriction enzymes. We tested two restriction enzymes that differ in the length of their recognition sites. ApeKI and PstI have a 5 bp and 6 bp recognition site, respectively. Furthermore, the ApeKI recognition site includes a 1 bp wobble. The utility of ApeKI for genome complexity reduction has already been reported for maize, where it was found to preferentially cut in the low-copy fraction of the genome [5]. It samples a large portion of the genome, as can be seen by the identification of 1,955,745 non redundant tags (loci) in this study, which is in stark contrast to the 252,879 loci identified using PstI. This ability to control the extent of genome complexity reduction is very useful, given that different applications will have different requirements when it comes to marker density. Each locus represents a potential marker, and the number that can be successfully converted to markers will be dependent on the population size, diversity and the Minor Allele Frequency (MAF) thresholds used for calling variants. However, with large enough population sizes and abundant diversity, we could envisage the vast majority of loci to contain a polymorphism. This was certainly the case in maize where they sequenced approximately 93 Mbp of the genome in a panel of 27 highly diverse inbred lines and found that 1 in every 44 bp harbored a polymorphism [4]. The choice between frequent and less frequent cutting enzymes is straight forward in many cases, e.g. population based studies in species with low linkage disequilibrium (LD) will require very high marker densities, whereas little benefit will be gained from such marker densities when working on within-family populations. When very little is known about the LD structure within a population, e.g. for specific breeding material, the choice can be more difficult. The availability of a reference genome will make the imputation of missing data possible when sequence coverage is low [18], however, this will not be possible in the many species without a reference genome. If each locus had an equal probability of being sequenced, then calculation of the sequencing depth necessary would be straight forward. However, each locus will be sequenced to varying degrees of coverage, and we have seen that this is highly correlated between samples (see figure S1). In order to predict the number of loci we could expect to genotype at different coverage levels, we randomly sampled reads to simulate different sequencing budgets and aligned them back to the set of consensus loci. At lower sequencing budgets we can expect to genotype more loci using PstI. However, as we begin to increase the sequencing budget, ApeKI quickly becomes a better alternative in terms of the numbers of loci we can genotype. However, if we require a greater sequencing depth at a relatively smaller number of loci, then it will be achieved at lower sequencing budgets using PstI. Certain applications may benefit from the rapid increase in the number of loci genotyped as we increase the sequencing budget. An example would be when developing genomic selection strategies in material with an unknown LD structure. After an initial round of genotyping it may become apparent that LD rapidly decays between markers and further genotyping is necessary. Further sequencing of ApeKI libraries will yield these additional markers, e.g. in our analysis with simulated sequencing budgets, going from 10 million to 20 million reads results in a 43% increase in the number of loci with a minimum sequencing depth of 5. The additional sequencing will have the added benefit of increasing the coverage of those loci already genotyped, which will be particularly beneficial for improving the accuracy of allele frequency calls. In the case of PstI the potential addition of markers will be limited by the much lower number of ‘sequence ready fragments’ generated through PstI library construction. Here, additional marker generation would require the creation of additional libraries with different restriction enzymes.

### Genome Wide Allele Frequency Fingerprints (GWAFFs)

GBS offers an excellent tool to generate Genome Wide Allele Frequency Fingerprints (GWAFFs) for a given population. We have demonstrated this by generating GWAFFs for perennial ryegrass varieties, which are synthetic populations derived from a poly-cross of multiple genotypes. We generated GWAFFs in four replicates of each of eight populations to evaluate reproducibility. PCA showed that the ‘sample replicates’ of each variety were indistinguishable based on GWAFFs, which highlights that our sampling protocol is reproducible. Sampling will be an important consideration for generating accurate GWAFFs of a population. In our case it involved germinating a large quantity of seed from each variety and taking consideration to pool approximately equal amounts of tissue from each germinated seedling. However, different applications and biological systems will require their own suitable sampling approach. The ‘sample replicates’ also allowed us to evaluate the reproducibility of our allele frequency calls. We performed pearson correlation analysis between the frequency of the variant allele in each sample. Not surprisingly, reproducibility improves as we increase coverage. At coverage levels greater than 20, we see very strong correlations (average r  = 0.91, stdev: 0.01, correlations using n = 1000), but even at relatively low coverage levels of between 5 and 10, we also see evidence of strong correlations (average r  = 0.67, stdev: 0.01, correlations using n = 1000).

Using a minimum coverage threshold of 5 we were able to distinguish varieties from one another with GWAFFs based on allele frequency calls at 20,942 and 10,958 SNP positions for ApeKI and PstI libraries, respectively. We evaluated our power to distinguish varieties with different SNP numbers, and it was clear that as the number of SNP positions for which we have allele frequency calls increases, our ability to distinguish between varieties improves correspondingly (Figure 4). GWAFFs based on allele frequencies at a very low number of SNP positions (e.g. 500), does not enable all varieties to be distinguished from one another.

In addition to distinguishing between varieties, GWAFFs also enable the partitioning of varieties into forages and turf types on the first principle component. This demonstrates the utility of using GWAFFs for analyzing variation on a population level. The usefulness of GWAFFs lies in their ability to measure the frequency of alleles in a population on a genome-wide scale, without the need to genotype a representative number of individuals from a population. Furthermore, like using GBS on single individuals, it incorporates discovery and characterization of genetic variation in a single step. This avoids any problems with SNP ascertainment bias. In this study we performed multiplexing of 32 barcoded samples in all our libraries, however, it is possible to increase the level of multiplexing. The degree of multiplexing that can be achieved will depend on (1) the level of throughput of the sequencing instrument, (2) the degree of genome complexity reduction, and (3) the level of coverage required to accurately call allele frequencies at the required number of SNP positions, given the complexity of the population. Current throughput on the Illumina HiSeq 2000 instrument is up to 1.5 billion single end reads per flow cell or up to 200 million reads per flow cell lane. However, throughput has been continually rising over the years so there is potential that this will increase further in the future. The two enzymes we tested in the current study differed greatly in the number of loci they generated for sequencing, and particularly in the case of PstI, there will be an opportunity to increase the number of populations that can be multiplexed. The variation in coverage across loci means that a percentage will have a relatively high coverage and can be used to generate a high quality GWAFF of the population. As more sequencing is done, more positions will be added to this high quality set. A useful approach for library creation may be to generate a library containing all populations to be multiplexed and sequencing this library on multiple lanes progressively. Using this strategy, the number of reads per sample can be monitored and additional ‘top-up’ libraries can be prepared to boost populations with low output. This will help to keep the variation in coverage between samples low, resulting in less missing values.

### Applications of GWAFFs

GAWFFs have numerous potential applications, including in the breeding of outbreeding species reliant on population-based breeding strategies. These species include forage legumes, forage grasses, oil palm and sugar beet. Many perennial ryegrass breeding programs use full-sib progeny selection in cultivar development, and accurate evaluation of many traits, including yield, must be done in swards [19]. It may be possible to use GWAFFs in combination with phenotypic data from the populations to develop genomic selection models. This is currently the focus of a project involving DLF-Trifolium and Aarhus University (http://www.forageselect.com/?lang=en). GWAFFs may also have application in breeding and genetic studies of allotetraploid species, where allele dosage is an important consideration. Approaches to determine allele dosage from array based SNP assays, which rely on signal intensity being proportional to allele dosage have already been evaluated [20]. Using a GBS strategy to develop GWAFFs would enable SNP discovery and accurate genotyping in a single step, thus ensuring the SNP panel is appropriate for the material under evaluation.

Another very interesting application of GWAFFs is in the monitoring of population structure over time. A good example of this is in the case of grasslands, which can undergo re-seeding infrequently. Over the years, it is likely that changes will occur in population allele frequencies, to a point where the population becomes significantly different from the original variety sown. These shifts in allele frequency may be the result of selection pressures not necessarily associated with improved productivity, and therefore the agricultural fitness of the grassland may decrease over time. Breeding varieties that can persist and produce stable grasslands for longer periods would be of significant benefit. GWAFFs could serve as an excellent tool to evaluate varietal persistence and relate changes in GWAFFs to changes in productivity. Other areas where monitoring shifts in genome-wide population allele frequencies may have a role include; monitoring plant pathogen populations over time or in response to different hosts, landscape genomics, and monitoring changes in genebank populations after multiplication. It may also be of assistance in reducing redundancy in under resourced genebanks, where duplicate accessions could be merged or one of the duplicates “archived” to reduce maintenance costs [21]. GWAFFs may be an economical way of evaluating diversity between accessions without the need for single plant genotyping.

GWAFFs may also have a function in varietal protection of outbreeding populations and allotetraploid species. Variety registration is based on the DUS system, which specifies that a variety must be distinct, stable, and uniform [22]. In the case of perennial ryegrass varieties, this involves the evaluation of numerous characteristics on 60 individual genotypes selected to represent the population. This is a huge amount of work because all new candidate varieties need to be evaluated against the complete back catalogue of registered varieties. Furthermore, as these characteristics are environmentally dependent, evaluation needs to be performed over multiple years. In this study we were able to distinguish varieties based on GWAFFs, and could see that our ability to distinguish between them improved as allele frequencies were obtained at greater numbers of SNP positions. GWAFFs may have a role to play in both determining distinctness, and evaluating stability of the variety through different submissions.

### Summary

This study highlights the ease at which current genotyping-by-sequencing strategies can be utilized to evaluate allele frequencies in population’s at large numbers of SNP positions across the genome. The resulting profiles have been termed Genome Wide Allele Frequency Fingerprints (GWAFFs), and we have shown that these fingerprints are reproducible and can be used to distinguish between plant populations. Even at current costs and throughput, using sequencing to directly evaluate populations on a genome wide scale is viable. GWAFFs should find many applications, from varietal development in outbreeding species right through to protection of plant breeders’ rights.

## Supporting Information

Figure S1 **Heatmaps of pearson correlation scores (between samples) of the number of reads mapping to the consensus loci for (A) PstI library, and (B) ApeKI library.** The correlations are based on coverage of loci containing SNP positions identified with a Minor Allele Frequency (MAF) of 5%. In the case of multiple SNPs per locus, only the first SNP in the locus was included, leaving over 24,000 data points for PstI, and over 350,000 data points for ApeKI.
(TIFF)

Figure S2 **Histogram showing the distribution of SNPs within reads for PstI and ApeKI libraries.** SNPs identified with a MAF threshold of 1% are shown on the left and those identified with a MAF threshold of 5% on the right.
(TIF)

Figure S3 **Barcharts showing the number of SNPs per loci for ApeKI (top) and PstI (bottom) libraries.**
(TIFF)

Methods S1 **Evaluation of alternative restriction enzymes tested for their potential to generate suitable GBS libraries for perennial ryegrass.**
(DOCX)

Results S1 **Tables showing the number of sequences per barcode and samples after demultiplexing both PstI and ApeKI GBS libraries (Bar Charts are also shown).** Also shown is a scatter plot matrix of the number of sequence per barcode across the eight lanes for the ApeKI GBS libraries.
(DOCX)

Results S2 **Scatterplots between ‘sampling replicates’ of allele frequencies calculated with different coverage thresholds.**
(DOCX)
