# Supplementary figures and images for: Genome Wide Allele Frequency Fingerprints (GWAFFs) of Populations via Genotyping by Sequencing
Source: PLoS One. 2013 Mar 4;8(3):e57438. doi: 10.1371/journal.pone.0057438 (PMC3587605; doi:10.1371/journal.pone.0057438)

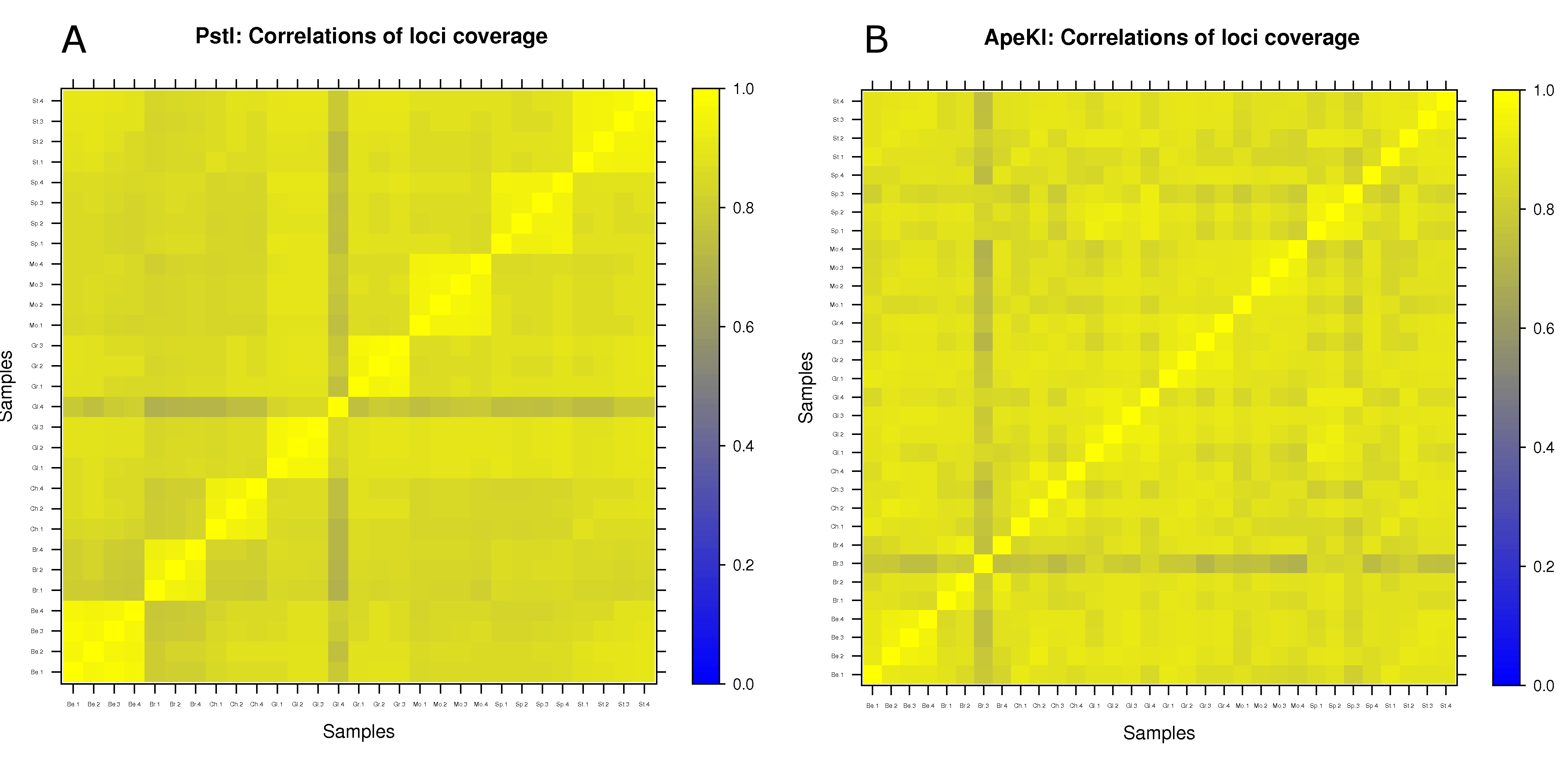

Supplement: Figure S1 — Heatmaps of pearson correlation scores (between samples) of the number of reads mapping to the consensus loci for (A) PstI library, and (B) ApeKI library. The correlations are based on coverage of loci containing SNP positions identified with a Minor Allele Frequency (MAF) of 5%. In the case of multiple SNPs per locus, only the first SNP in the locus was included, leaving over 24,000 data points for PstI, and over 350,000 data points for ApeKI. (TIFF) [file pone.0057438.s001.tif]

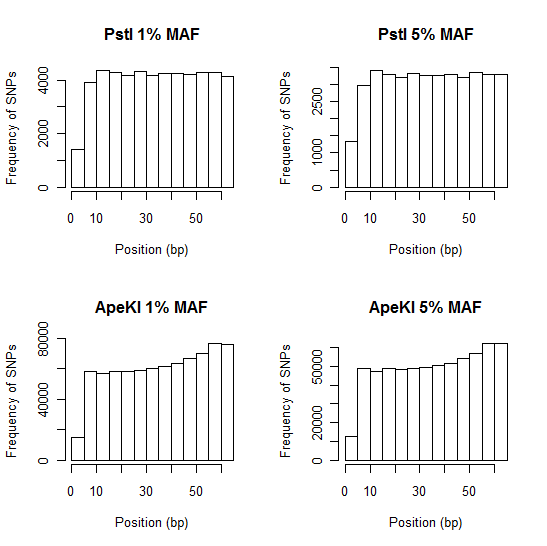

Supplement: Figure S2 — Histogram showing the distribution of SNPs within reads for PstI and ApeKI libraries. SNPs identified with a MAF threshold of 1% are shown on the left and those identified with a MAF threshold of 5% on the right. (TIF) [file pone.0057438.s002.tif]

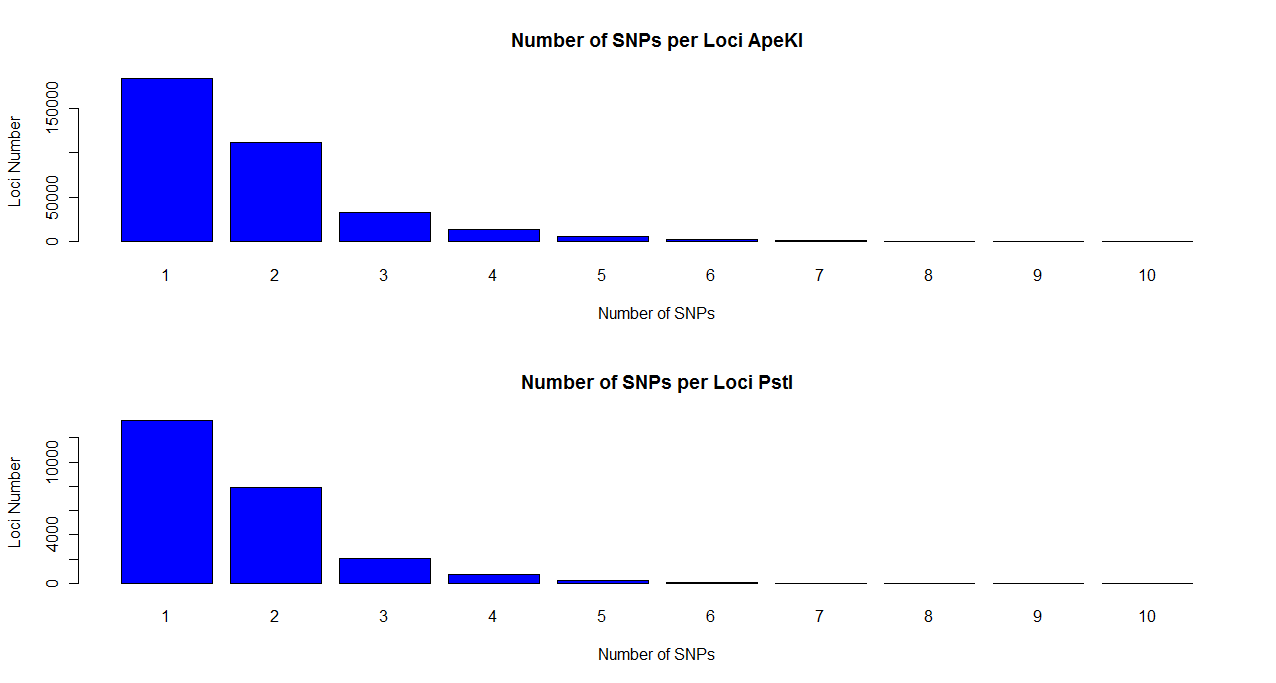

Supplement: Figure S3 — Barcharts showing the number of SNPs per loci for ApeKI (top) and PstI (bottom) libraries. (TIFF) [file pone.0057438.s003.tiff]
