# Supplementary material for: Genome Wide Allele Frequency Fingerprints (GWAFFs) of Populations via Genotyping by Sequencing
Source: PLoS One. 2013 Mar 4;8(3):e57438. doi: 10.1371/journal.pone.0057438 (PMC3587605; doi:10.1371/journal.pone.0057438)
Supplement: Methods S1 — Evaluation of alternative restriction enzymes tested for their potential to generate suitable GBS libraries for perennial ryegrass. (DOCX) [file pone.0057438.s004.docx]

**Supplemental MethodsS1**

**Evaluating restriction enzymes for genotyping by sequencing in perennial ryegrass.**

Four restriction enzymes were selected for evaluation after performing *in silico* digestions of the maize genome (Table S1). This was performed to obtain an insight into the number of fragments that would potentially be available for sequencing after digestion with different enzymes. While this does not take into account factors such as methylation, it does provide a starting point for estimating the number of loci available for sequencing. Libraries were created with these four enzymes to evaluate library size distribution after adaptor ligation and library amplification. The resulting libraries were run on a DNA1000 Agilent Bioanalyzer chip for the separation and sizing of dsDNA fragments between 25 and 1000bp (Figure S1). In Figure S1(A), AgeI, there is an adaptor dimer at ~128bp which we were unable to remove after optimization of adaptor to DNA ratio. In Figure S1(B), EcoRI, there is a lot of clear individuals peaks, indicative of highly repetitive fragments. ApeKI and PstI (Figure S1(C and D)), show restriction profiles more suitable for GBS. The size profile of the ApeKI library is ideal as the greatest portion of fragments are larger than 200bp (inserts greater than 80bp). The profile of the PstI library has a relatively larger portion of fragments smaller than 200bp (inserts less than 80bp).

Table S1(1). Summary of enzymes used for creation of GBS libraries and results of in silico digestions of the maize genome.

| Enzyme name | Recognition Site | Fragments ≥ 100bp and ≤ 1KB | No. GBS tags |
| --- | --- | --- | --- |
| PstI | CTGCA^G | 136,644 | 273,288 |
| ApekI | G^CWGC | 2,203,708 | 4,407,416 |
| AgeI | A^CCGGT | 30,410 | 60,820 |
| EcoRI | G^AATTC | 104,428 | 208,856 |


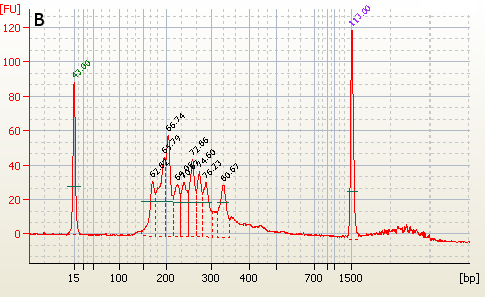
**
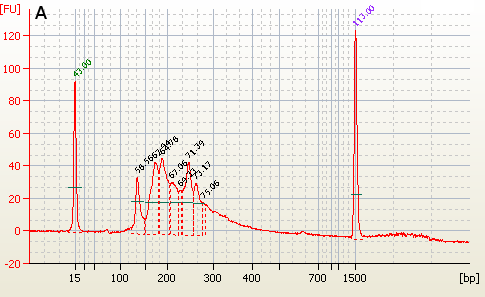
**


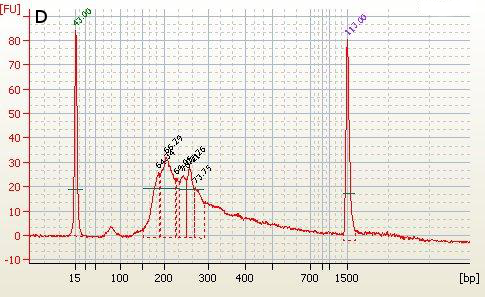
**
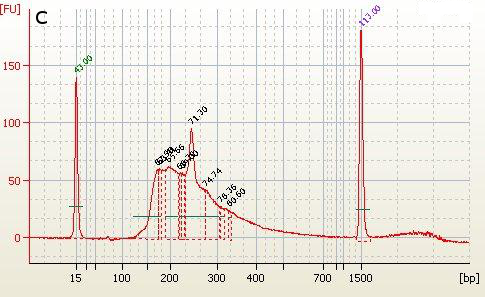
**

Figure S1: Electropherograms of GBS libraries generated using different restriction enzymes. A: AgeI, B: EcoRI, C: PstI, D: ApekI. These are final libraries after adaptor ligation and amplification. Size in bp is shown on the horizontal axis of each plot. Peaks at 15bp and 1500bp are internal standards.
