# Supplementary material for: Genome Wide Allele Frequency Fingerprints (GWAFFs) of Populations via Genotyping by Sequencing
Source: PLoS One. 2013 Mar 4;8(3):e57438. doi: 10.1371/journal.pone.0057438 (PMC3587605; doi:10.1371/journal.pone.0057438)
Supplement: Results S1 — Tables showing the number of sequences per barcode and samples after demultiplexing both PstI and ApeKI GBS libraries (Bar Charts are also shown). Also shown is a scatter plot matrix of the number of sequence per barcode across the eight lanes for the ApeKI GBS libraries. (DOCX) [file pone.0057438.s005.docx]

**Supplemental Results S1**

Tables showing the number of sequences per barcode and samples after demultiplexing both PstI and ApeKI GBS libraries (Bar Charts are also shown). A scatter plot matrix of number of sequence per barcode across the eight lanes is shown for the ApeKI GBS libraries.

Table S1(A): The number of sequences generated for each of the 32 barcoded samples in each of the eight lanes. Each lane sequenced an ApeKI GBS library corresponding to one of eight varieties. The same set of 32 barcodes was used in each lane.

| Barcode Name | Barcode Sequence | **Greenway** | **Beatrice** | **Bronzyn** | **Chardine** | **Sponsor** | **Stolon** | **Glenveagh** | **Mongita** |
| --- | --- | --- | --- | --- | --- | --- | --- | --- | --- |
| BCA1 | ctcc | 391792 | 469545 | 445919 | 496812 | 370306 | 341049 | 351414 | 428725 |
| BCA4 | gtct | 577014 | 582128 | 712598 | 477543 | 645221 | 405902 | 612769 | 426099 |
| BCA6 | gcgt | 654055 | 721752 | 708241 | 763903 | 636011 | 759147 | 551741 | 724540 |
| BCA8 | cgat | 656323 | 717624 | 427515 | 294817 | 783972 | 765331 | 687653 | 837666 |
| BCA9 | cttga | 758753 | 832754 | 600537 | 778548 | 854391 | 634149 | 652167 | 767711 |
| BCA10 | tcacc | 539888 | 643070 | 570217 | 781093 | 462790 | 483814 | 464453 | 587792 |
| BCA12 | acaaa | Failed | 749600 | 1025436 | 968546 | 641916 | 704284 | 798120 | 813973 |
| BCA13 | ttctc | 661080 | 699205 | 721886 | 735858 | 636719 | 754095 | 595149 | 708918 |
| BCA15 | gtatt | 1083576 | 1027224 | 1261853 | 1055633 | 1241098 | 1033411 | 1282801 | 1148481 |
| BCA16 | ctgta | 617321 | 626246 | 603397 | 586979 | 636297 | 384213 | 588316 | 498656 |
| BCA18 | actat | 966376 | 1255294 | 1239759 | 1303018 | 1091525 | 1147332 | 1001049 | 1287951 |
| BCA20 | ccagct | 758227 | 768460 | 522660 | 652744 | 755687 | 769008 | 672624 | 819928 |
| BCA21 | ttcaga | 1135244 | 1205350 | 1008370 | 953725 | 1274831 | 987508 | 781335 | 1216570 |
| BCA22 | taggaa | 945188 | 987412 | 909326 | 1111719 | 773760 | 761844 | 775318 | 943394 |
| BCA24 | ccacaa | 648773 | 668590 | 729702 | 824779 | 660113 | 528448 | 539525 | 657660 |
| BCA25 | cgtcaa | 859215 | 733498 | 955391 | 734698 | 890765 | 728690 | 804850 | 859628 |
| BCA27 | gagata | 914923 | 611457 | 848387 | 717204 | 984461 | 641636 | 1036731 | 421188 |
| BCA28 | atgcct | 619625 | 717404 | 810741 | 926159 | 701981 | 676285 | 662146 | 833819 |
| BCA30 | cttgctt | 735204 | 885494 | 846055 | 894439 | 718798 | 743938 | 684633 | 674067 |
| BCA31 | atgaaac | 1181833 | 1420975 | 1171444 | 1346766 | 1072764 | 960823 | 907231 | 1089001 |
| BCA32 | aaaagtt | 1322971 | 1332761 | 746671 | 1033081 | 1350487 | 1263490 | 1190991 | 1486197 |
| BCA33 | gaattca | 1151314 | 1215867 | 1011271 | 1094783 | 1244748 | 864079 | 791024 | 1210809 |
| BCA34 | gaacttc | 1232167 | 1100146 | 1185123 | 1500639 | 873018 | 1095750 | 1008807 | 1178378 |
| BCA35 | ggaccta | 966796 | 901382 | 999199 | 1091587 | 723622 | 659741 | 825869 | 972925 |
| BCA39 | ACGACTAC | 702128 | 501804 | 683392 | 455416 | 734761 | 382331 | 701473 | 321967 |
| BCA40 | AAGGATGC | 872766 | 878993 | 970367 | 1081500 | 918194 | 826605 | 741668 | 1057609 |
| BCA41 | TAGCATGC | 544065 | 749300 | 757147 | 815862 | 630389 | 613201 | 542898 | 754726 |
| BCA42 | TAGGAAGC | 592328 | 800858 | 762220 | 783030 | 634315 | 533049 | 632596 | 677220 |
| BCA43 | TAGGCCAT | 688006 | 739627 | 552576 | 536242 | 792602 | 704271 | 680192 | 827515 |
| BCA44 | TGCAAGGA | 815042 | 804971 | 673865 | 620623 | 920835 | 709636 | 710679 | 750735 |
| BCA45 | TGGTACGT | 781302 | 858612 | 952865 | 1028975 | 720591 | 836723 | 824598 | 903234 |
| BCA47 | CCGGATAT | 732274 | 670838 | 843196 | 830634 | 566980 | 482032 | 609959 | 697567 |
|  | **matched** | 25105569 | 26878241 | 26257326 | 27277355 | 25943948 | 23181815 | 23710779 | 26584649 |
|  | **total** | 25664064 | 27231668 | 26576940 | 27619954 | 26252419 | 24659032 | 24007947 | 26904063 |
|  | **unmatched** | 558495 | 353427 | 319614 | 342599 | 308471 | 1477217 | 297168 | 319414 |
|  | **Mean** | 809857.0645 | 839945.03 | 820541.44 | 852417.34 | 810748.38 | 724431.7 | 740961.8438 | 830770.281 |
|  | **STDEV** | 230612.1759 | 239764.95 | 223547.98 | 272997.97 | 237526.03 | 226173.4 | 200174.4034 | 276542.009 |
|  | **Coefficient Variation** | 0.284756639 | 0.2854531 | 0.2724396 | 0.3202633 | 0.2929713 | 0.312208 | 0.270154806 | 0.33287422 |
|  | **Minimum** | 391792 | 469545 | 427515 | 294817 | 370306 | 341049 | 351414 | 321967 |
|  | **Maximum** | 1322971 | 1420975 | 1261853 | 1500639 | 1350487 | 1263490 | 1282801 | 1486197 |

Figure S1(A): Barchart showing the performance of each barcode across the eight varieties for the ApeKI GBS libraries.


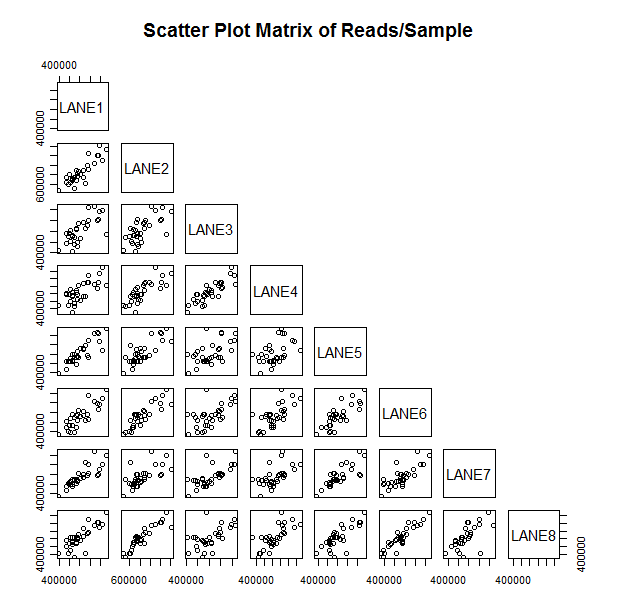


Figure S1(B): Scatter plot matrix on the number of sequences yielded by each barcoded sample across the eight varieties. Correlation indicates similar efficiencies in barcode performance across flow cell lanes.

Table SD1(B): The number of sequences generated for each of the 32 barcoded samples in the PstI GBS library. A single lane was sequenced that contained all eight varieties, each with four ‘sampling replicates’.

| Barcode Name | Barcode Sequence | Reads |
| --- | --- | --- |
| AD-1 | TGATTCATT | 14041770 |
| AD-2 | ACGTCCGGA | 4742897 |
| AD-3 | GAAGCGCCT | 4772973 |
| AD-4 | CTTAATTGT | 3741557 |
| AD-5 | AGCCGGACT | 4764232 |
| AD-6 | CAGCTCCA | 4353788 |
| AD-7 | TTCGGAGT | 3758473 |
| AD-8 | CCACACTG | 3139985 |
| AD-9 | GCAAGAAT | 4461754 |
| AD-10 | AATAGCAG | 8710 |
| AD-11 | TGGCCAA | 3926463 |
| AD-12 | GAAGCCT | 2345093 |
| AD-13 | GGATTCA | 7527702 |
| AD-14 | CTGGACA | 5859793 |
| AD-15 | GATTACA | 4384177 |
| AD-16 | AGCTGA | 5715202 |
| AD-17 | GACTCT | 4834953 |
| AD-18 | TTGACA | 4201583 |
| AD-19 | TGTGCA | 2826780 |
| AD-20 | GTTCCA | 2448098 |
| AD-21 | ACCTG | 169659 |
| AD-22 | TCGAA | 4792031 |
| AD-23 | ATCGA | 5826715 |
| AD-24 | CATCG | 4248430 |
| AD-25 | TCACG | 2583759 |
| AD-26 | CTGCA | 533303 |
| AD-27 | ATCA | 2692403 |
| AD-28 | GCTT | 2319031 |
| AD-29 | CGAG | 3298915 |
| AD-30 | CATA | 2939502 |
| AD-31 | TGCT | 2749887 |
| AD-32 | ACGG | 2772379 |
|  | **matched** | 126781997 |
|  | **total** | 126781997 |
|  | **unmatched** | 3666314 |
|  | **Mean** | 3961937.406 |
|  | **STDEV** | 2461707.772 |
|  | **Coefficient Var** | 0.62133939 |
|  | **Minimum** | 14041770 |
|  | **Maximum** | 8710 |

Figure S1(C): Barchart showing the performance of each of the 32 barcodes for the PstI GBS libraries.
