# Supplementary material for: Genome Wide Allele Frequency Fingerprints (GWAFFs) of Populations via Genotyping by Sequencing
Source: PLoS One. 2013 Mar 4;8(3):e57438. doi: 10.1371/journal.pone.0057438 (PMC3587605; doi:10.1371/journal.pone.0057438)

**Supplemental Results S2**

To investigate the effect of coverage on reproducibility, we placed SNPs into three groups depending on the coverage at this position falling into one of three ranges (5≤X<10, 10≤X<20, X≥20) for each of the four ‘sample replicates’. We randomly sampled 1000 SNP positions from each range to ensure an equal number of data points between comparisons. Scatter plots of all the possible pairwise comparisons for the varieties Bronzyn, Chardin, Glenveagh, Greenway, Monigta, Sponsor, and Stolon are shown in the following figures (in that order). (A) 5≤X<10, (B)10≤X<20, and (C) X≥20. Least squares regression line is shown by solid green line, and Loess smooth is shown by broken red line.


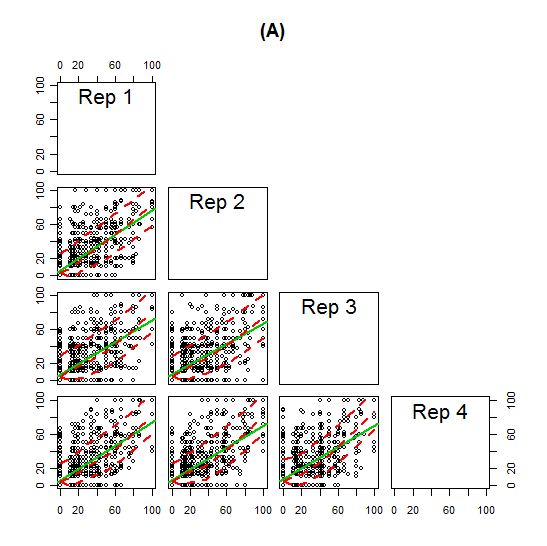


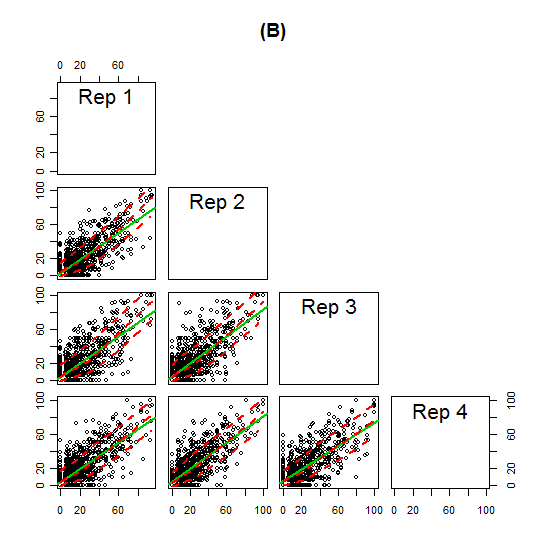

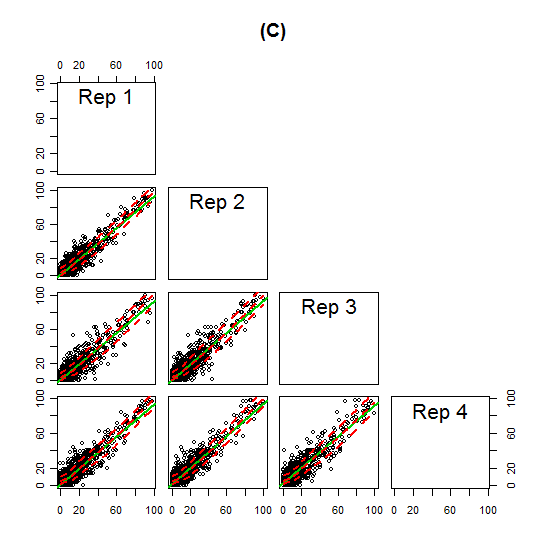


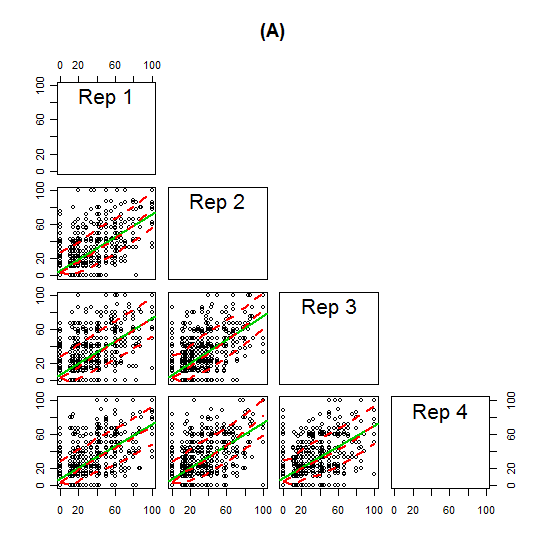

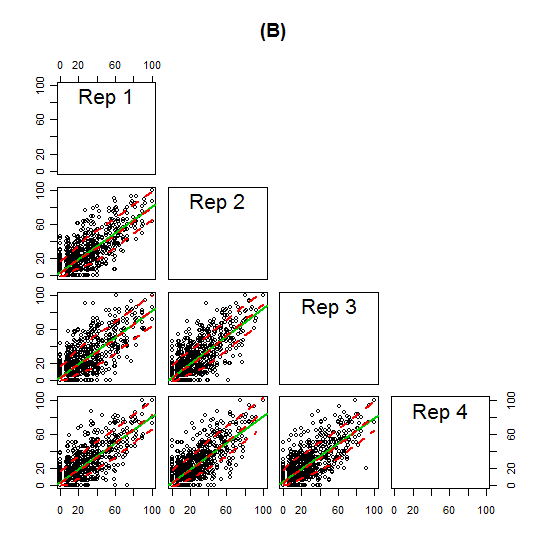

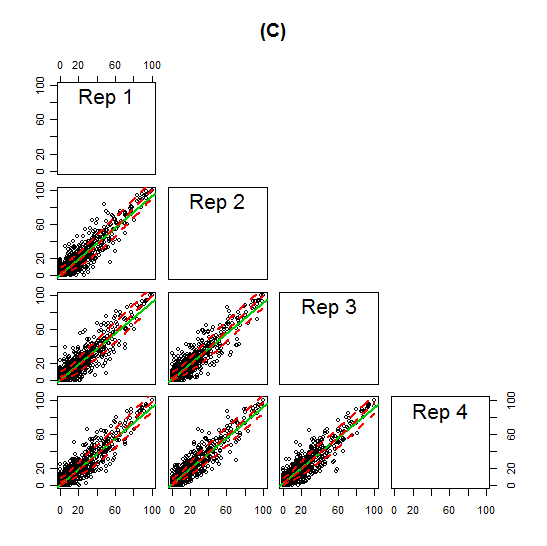

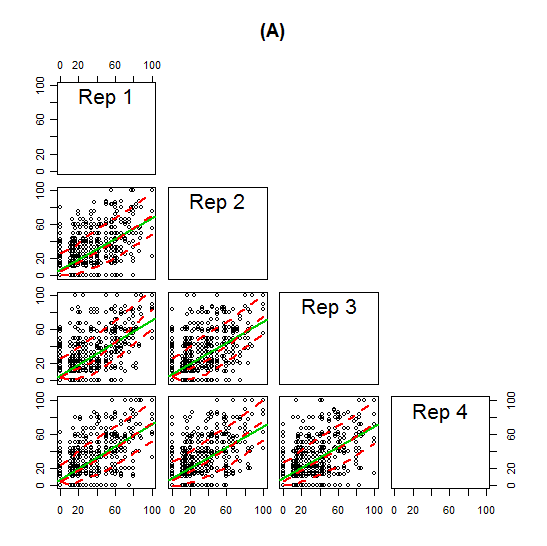

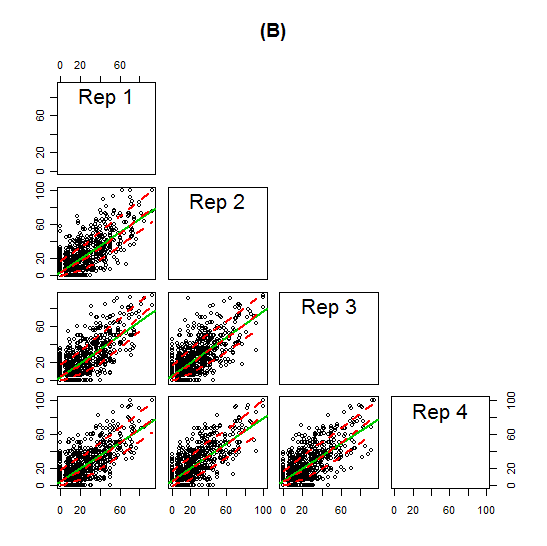

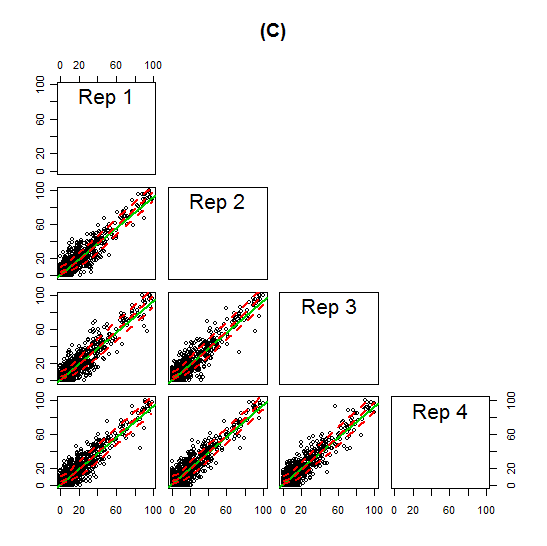

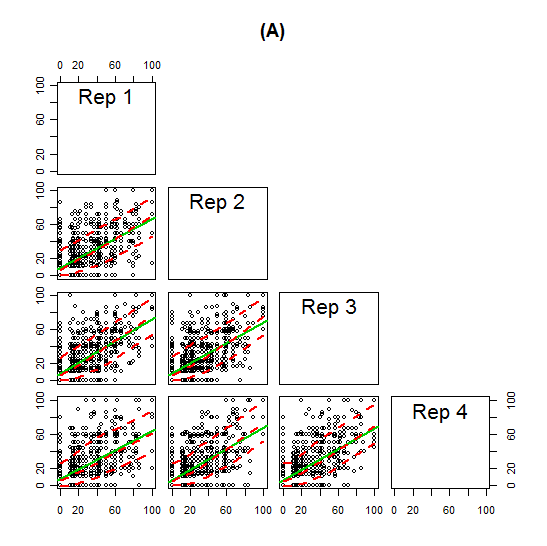

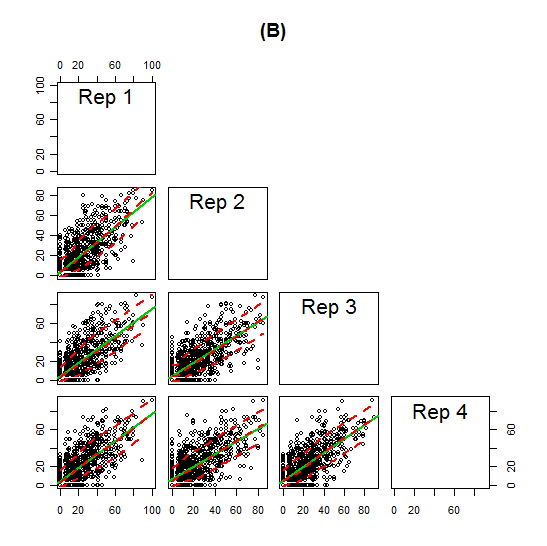

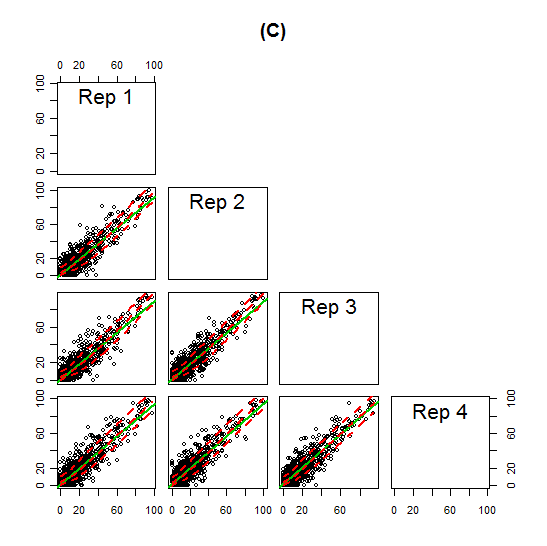

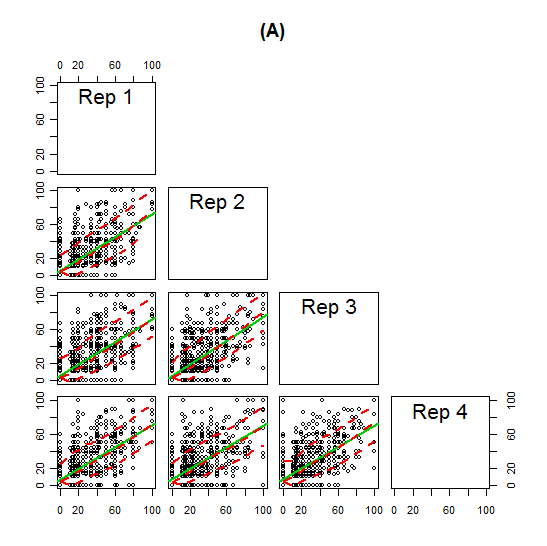

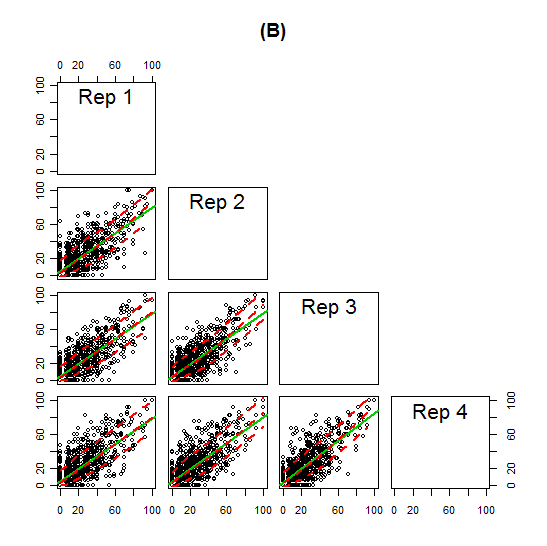

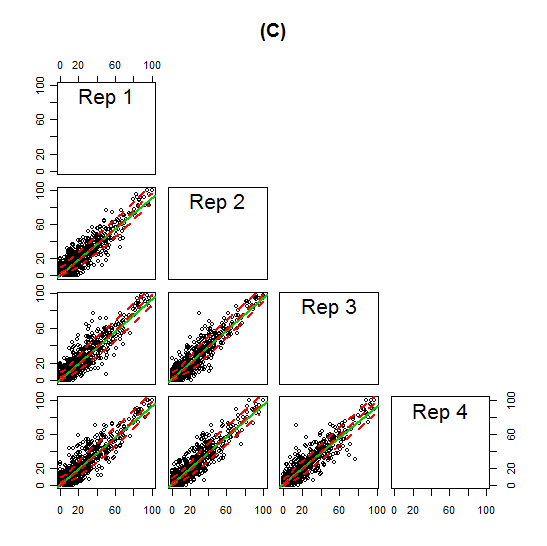

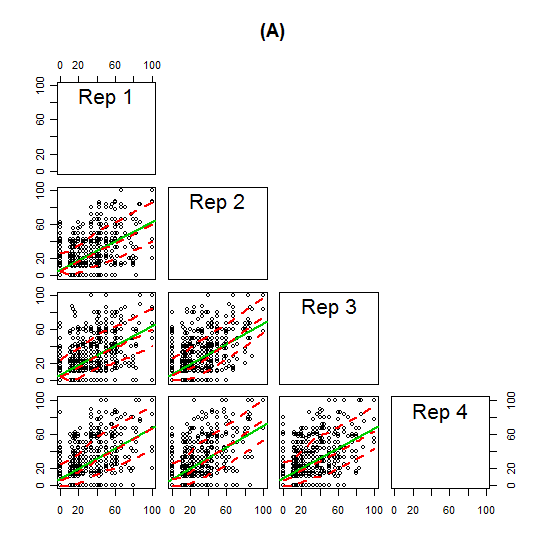

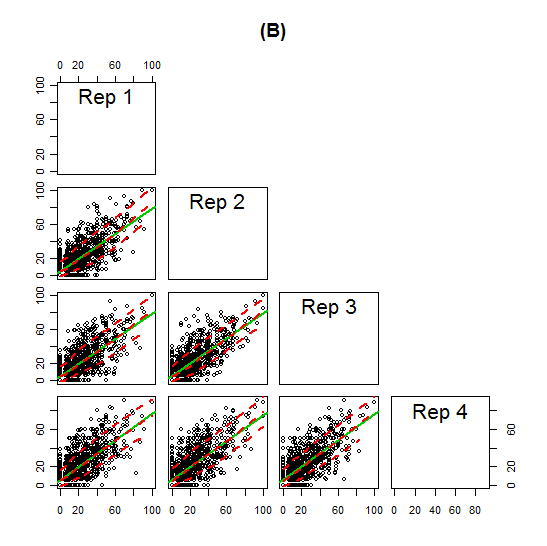

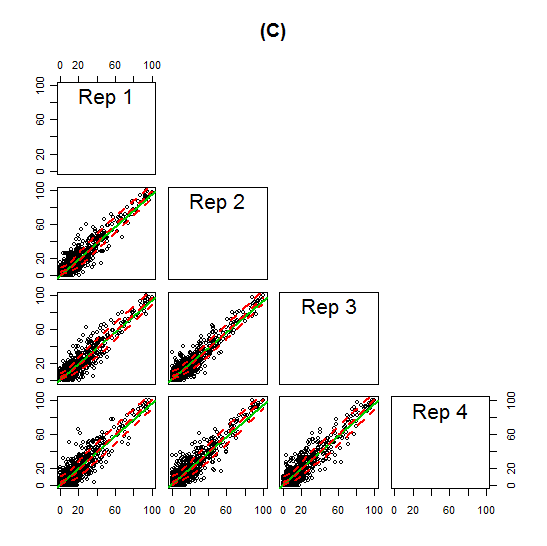

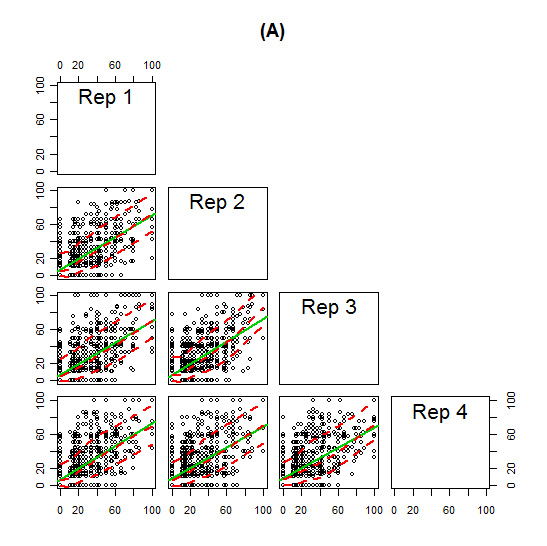

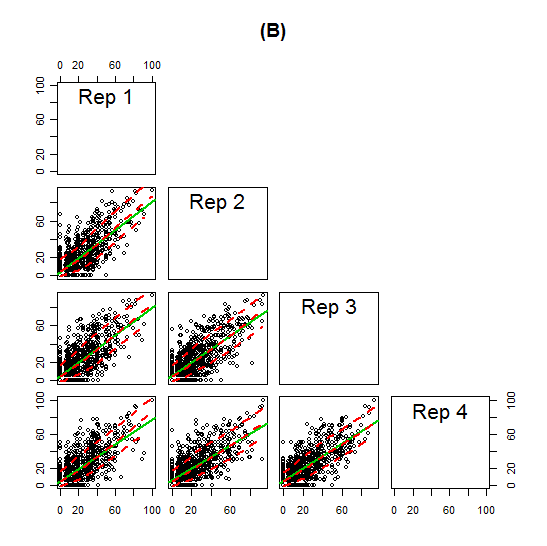

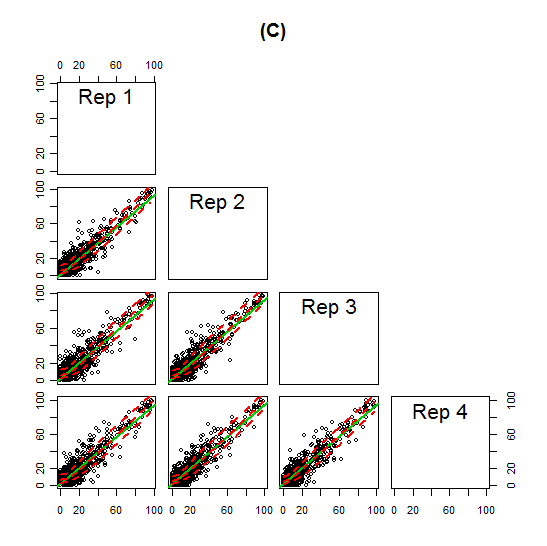

Supplement: Results S2 — Scatterplots between ‘sampling replicates’ of allele frequencies calculated with different coverage thresholds. (DOCX) [file pone.0057438.s006.docx]
